# Supplementary material for: Unveiling the Bioactive Potential of the Invasive Jellyfish Phyllorhiza punctata Through Integrative Transcriptomic and Proteomic Analyses
Source: Biomolecules. 2025 Aug 4;15(8):1121. doi: 10.3390/biom15081121 (PMC12383608; doi:10.3390/biom15081121)
Supplement: Supplementary file 1 [file biomolecules-15-01121-s001.zip › Suplementary Material/FileS3/PEPFOLD4/XP_065053185_13658/clusters.html]

 PEP-FOLD clusters


Download archive of all models  here   

|  |  |  |
| --- | --- | --- |
| # | file | sOPEP |
| # cluster 1 - Size: 1 |  |  |
|  | XP\_065053185\_13658-model1 | -26.2938 |
| # cluster 2 - Size: 4 |  |  |
|  | XP\_065053185\_13658-model2 | -23.6215 |
|  | XP\_065053185\_13658-model2.1 | -17.2389 |
|  | XP\_065053185\_13658-model2.2 | -17.1623 |
|  | XP\_065053185\_13658-model2.3 | -16.7032 |
| # cluster 3 - Size: 2 |  |  |
|  | XP\_065053185\_13658-model3 | -20.7075 |
|  | XP\_065053185\_13658-model3.1 | -18.6697 |
| # cluster 4 - Size: 1 |  |  |
|  | XP\_065053185\_13658-model4 | -20.6227 |
| # cluster 5 - Size: 3 |  |  |
|  | XP\_065053185\_13658-model5 | -20.582 |
|  | XP\_065053185\_13658-model5.1 | -18.6671 |
|  | XP\_065053185\_13658-model5.2 | -17.0735 |
| # cluster 6 - Size: 13 |  |  |
|  | XP\_065053185\_13658-model6 | -20.3931 |
|  | XP\_065053185\_13658-model6.1 | -20.0945 |
|  | XP\_065053185\_13658-model6.2 | -19.8618 |
|  | XP\_065053185\_13658-model6.3 | -19.3792 |
|  | XP\_065053185\_13658-model6.4 | -19.3342 |
|  | XP\_065053185\_13658-model6.5 | -18.9964 |
|  | XP\_065053185\_13658-model6.6 | -18.5846 |
|  | XP\_065053185\_13658-model6.7 | -18.436 |
|  | XP\_065053185\_13658-model6.8 | -17.9807 |
|  | XP\_065053185\_13658-model6.9 | -17.6839 |
|  | XP\_065053185\_13658-model6.10 | -16.7736 |
|  | XP\_065053185\_13658-model6.11 | -15.907 |
|  | XP\_065053185\_13658-model6.12 | -15.0585 |
| # cluster 7 - Size: 1 |  |  |
|  | XP\_065053185\_13658-model7 | -20.2481 |
| # cluster 8 - Size: 2 |  |  |
|  | XP\_065053185\_13658-model8 | -20.1593 |
|  | XP\_065053185\_13658-model8.1 | -13.9192 |
| # cluster 9 - Size: 1 |  |  |
|  | XP\_065053185\_13658-model9 | -20.1203 |
| # cluster 10 - Size: 2 |  |  |
|  | XP\_065053185\_13658-model10 | -19.8046 |
|  | XP\_065053185\_13658-model10.1 | -17.5835 |
| # cluster 11 - Size: 5 |  |  |
|  | XP\_065053185\_13658-model11 | -19.4872 |
|  | XP\_065053185\_13658-model11.1 | -19.4214 |
|  | XP\_065053185\_13658-model11.2 | -19.0448 |
|  | XP\_065053185\_13658-model11.3 | -18.193 |
|  | XP\_065053185\_13658-model11.4 | -15.0583 |
| # cluster 12 - Size: 1 |  |  |
|  | XP\_065053185\_13658-model12 | -19.4415 |
| # cluster 13 - Size: 2 |  |  |
|  | XP\_065053185\_13658-model13 | -19.16 |
|  | XP\_065053185\_13658-model13.1 | -16.6478 |
| # cluster 14 - Size: 2 |  |  |
|  | XP\_065053185\_13658-model14 | -18.7497 |
|  | XP\_065053185\_13658-model14.1 | -16.5849 |
| # cluster 15 - Size: 2 |  |  |
|  | XP\_065053185\_13658-model15 | -18.5165 |
|  | XP\_065053185\_13658-model15.1 | -16.7429 |
| # cluster 16 - Size: 4 |  |  |
|  | XP\_065053185\_13658-model16 | -18.379 |
|  | XP\_065053185\_13658-model16.1 | -18.2433 |
|  | XP\_065053185\_13658-model16.2 | -17.8378 |
|  | XP\_065053185\_13658-model16.3 | -15.109 |
| # cluster 17 - Size: 2 |  |  |
|  | XP\_065053185\_13658-model17 | -18.2767 |
|  | XP\_065053185\_13658-model17.1 | -17.6774 |
| # cluster 18 - Size: 1 |  |  |
|  | XP\_065053185\_13658-model18 | -18.2734 |
| # cluster 19 - Size: 1 |  |  |
|  | XP\_065053185\_13658-model19 | -18.1838 |
| # cluster 20 - Size: 8 |  |  |
|  | XP\_065053185\_13658-model20 | -17.9182 |
|  | XP\_065053185\_13658-model20.1 | -17.6561 |
|  | XP\_065053185\_13658-model20.2 | -16.3349 |
|  | XP\_065053185\_13658-model20.3 | -15.7181 |
|  | XP\_065053185\_13658-model20.4 | -15.6049 |
|  | XP\_065053185\_13658-model20.5 | -14.0093 |
|  | XP\_065053185\_13658-model20.6 | -12.601 |
|  | XP\_065053185\_13658-model20.7 | -11.6996 |
| # cluster 21 - Size: 1 |  |  |
|  | XP\_065053185\_13658-model21 | -17.8428 |
| # cluster 22 - Size: 1 |  |  |
|  | XP\_065053185\_13658-model22 | -17.7613 |
| # cluster 23 - Size: 1 |  |  |
|  | XP\_065053185\_13658-model23 | -17.5221 |
| # cluster 24 - Size: 1 |  |  |
|  | XP\_065053185\_13658-model24 | -16.9426 |
| # cluster 25 - Size: 1 |  |  |
|  | XP\_065053185\_13658-model25 | -16.9038 |
| # cluster 26 - Size: 1 |  |  |
|  | XP\_065053185\_13658-model26 | -16.812 |
| # cluster 27 - Size: 1 |  |  |
|  | XP\_065053185\_13658-model27 | -16.6998 |
| # cluster 28 - Size: 1 |  |  |
|  | XP\_065053185\_13658-model28 | -16.4472 |
| # cluster 29 - Size: 1 |  |  |
|  | XP\_065053185\_13658-model29 | -16.4388 |
| # cluster 30 - Size: 1 |  |  |
|  | XP\_065053185\_13658-model30 | -16.4237 |
| # cluster 31 - Size: 2 |  |  |
|  | XP\_065053185\_13658-model31 | -16.3424 |
|  | XP\_065053185\_13658-model31.1 | -16.3206 |
| # cluster 32 - Size: 1 |  |  |
|  | XP\_065053185\_13658-model32 | -16.2975 |
| # cluster 33 - Size: 1 |  |  |
|  | XP\_065053185\_13658-model33 | -16.1936 |
| # cluster 34 - Size: 1 |  |  |
|  | XP\_065053185\_13658-model34 | -15.9903 |
| # cluster 35 - Size: 2 |  |  |
|  | XP\_065053185\_13658-model35 | -15.9024 |
|  | XP\_065053185\_13658-model35.1 | -14.4778 |
| # cluster 36 - Size: 2 |  |  |
|  | XP\_065053185\_13658-model36 | -15.6372 |
|  | XP\_065053185\_13658-model36.1 | -15.5956 |
| # cluster 37 - Size: 3 |  |  |
|  | XP\_065053185\_13658-model37 | -15.5356 |
|  | XP\_065053185\_13658-model37.1 | -15.3181 |
|  | XP\_065053185\_13658-model37.2 | -13.3387 |
| # cluster 38 - Size: 1 |  |  |
|  | XP\_065053185\_13658-model38 | -15.0361 |
| # cluster 39 - Size: 3 |  |  |
|  | XP\_065053185\_13658-model39 | -15.0184 |
|  | XP\_065053185\_13658-model39.1 | -14.0357 |
|  | XP\_065053185\_13658-model39.2 | -11.9679 |
| # cluster 40 - Size: 2 |  |  |
|  | XP\_065053185\_13658-model40 | -14.9225 |
|  | XP\_065053185\_13658-model40.1 | -14.8966 |
| # cluster 41 - Size: 1 |  |  |
|  | XP\_065053185\_13658-model41 | -14.9198 |
| # cluster 42 - Size: 1 |  |  |
|  | XP\_065053185\_13658-model42 | -14.4074 |
| # cluster 43 - Size: 1 |  |  |
|  | XP\_065053185\_13658-model43 | -14.2833 |
| # cluster 44 - Size: 1 |  |  |
|  | XP\_065053185\_13658-model44 | -14.2605 |
| # cluster 45 - Size: 1 |  |  |
|  | XP\_065053185\_13658-model45 | -14.109 |
| # cluster 46 - Size: 1 |  |  |
|  | XP\_065053185\_13658-model46 | -13.6553 |
| # cluster 47 - Size: 1 |  |  |
|  | XP\_065053185\_13658-model47 | -13.3853 |
| # cluster 48 - Size: 2 |  |  |
|  | XP\_065053185\_13658-model48 | -13.2867 |
|  | XP\_065053185\_13658-model48.1 | -12.4314 |
| # cluster 49 - Size: 1 |  |  |
|  | XP\_065053185\_13658-model49 | -13.2319 |
| # cluster 50 - Size: 1 |  |  |
|  | XP\_065053185\_13658-model50 | -12.7996 |
| # cluster 51 - Size: 1 |  |  |
|  | XP\_065053185\_13658-model51 | -12.3354 |
| # cluster 52 - Size: 1 |  |  |
|  | XP\_065053185\_13658-model52 | -12.2921 |
| # cluster 53 - Size: 1 |  |  |
|  | XP\_065053185\_13658-model53 | -11.3347 |
|  |  |  |
